# Cerebrotendinous xanthomatosis: a comprehensive review of pathogenesis, clinical manifestations, diagnosis, and management

**DOI:** 10.1186/s13023-014-0179-4

**Published:** 2014-11-26

**Authors:** Shuke Nie, Guiqin Chen, Xuebing Cao, Yunjian Zhang

**Affiliations:** Department of Neurology, Union Hospital, Tongji Medical College, Huazhong University of Science and Technology, 1277 Jiefang Avenue, Wuhan, 430022 China

**Keywords:** Cerebrotendinous xanthomatosis, Xanthoma, *CYP27A1*, Sterol 27-hydroxylase, Chenodeoxycholic acid, Cholic acid

## Abstract

Cerebrotendinous xanthomatosis (CTX) OMIM#213700 is a rare autosomal-recessive lipid storage disease caused by mutations in the *CYP27A1* gene; this gene codes for the mitochondrial enzyme sterol 27-hydroxylase, which is involved in bile acid synthesis. The *CYP27A1* gene is located on chromosome 2q33-qter and contains nine exons. A *CYP27A1* mutation leads to decreased synthesis of bile acid, excess production of cholestanol, and consequent accumulation of cholestanol in tissues. Currently there is no consensus on the prevalence of CTX, one estimate being <5/100,000 worldwide. The prevalence of CTX due to the *CYP27A1* mutation *R362C* alone is approximately 1/50,000 in Caucasians. Patients with CTX have an average age of 35 years at the time of diagnosis and a diagnostic delay of 16 years. Clinical signs and symptoms include adult-onset progressive neurological dysfunction (i.e., ataxia, dystonia, dementia, epilepsy, psychiatric disorders,peripheral neuropathy, and myopathy) and premature non-neurologic manifestations (i.e., tendon xanthomas, childhood-onset cataracts, infantile-onset diarrhea, premature atherosclerosis, osteoporosis, and respiratory insufficiency). Juvenile cataracts, progressive neurologic dysfunction, and mild pulmonary insufficiency are unique symptoms that distinguish CTX from other lipid storage disorders including familial dysbetalipoproteinemia, homozygous familial hypercholesterolemia, and sitosterolemia, all of which might also present with xanthomas and cardiovascular diseases. Brain magnetic resonance imaging (MRI) shows bilateral lesions in the dentate nucleus of the cerebellum and mild white matter lesions. The classical symptoms and signs, namely elevated levels of cholestanol and bile alcohols in serum and urine, brain MRI, and the mutation in the *CYP27A1* gene confirm the diagnosis of CTX. Early diagnosis and long-term treatment with chenodeoxycholic acid (750 mg/d) improve neurological symptoms and contribute to a better prognosis.

## Introduction

### Definition, history, epidemiology

Cerebrotendinous xanthomatosis (CTX; OMIM#213700) is a rare autosomal-recessive inborn disorder of bile acid metabolism due to mutations in the *CYP27A1* gene(OMIM *606530) located on chromosome 2q33-qter, leading to increased deposition of cholesterol and cholestanol in multiple tissues [1]. Since Bogaert’s first report in 1937 of a case of CTX, more than several hundred cases have been reported worldwide [2,3]. CTX may have a higher prevalence than usually recognized. There are no consensus data on the prevalence of CTX, the estimated rate being <5/100,000 worldwide [4]. The prevalence of this disease varies with country and ethnic group; the prevalence of CTX due to the *CYP27A1* mutation *R362C* alone is 1/800,000 individuals in Spain and is approximately 1/50,000 in Caucasians [4,5].

### Etiology and biochemical pathogenesis

**Figure 1:**
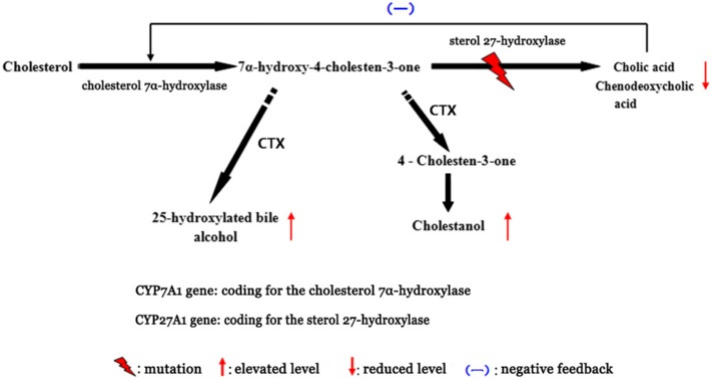
**Metabolic pathway involved in cerebrotendinous xanthomatosis (CTX; modified from Chales and Bjorkhem** **[** 10 **,** 74 **]** **).** Due to a mutation in the *CYP27A1* gene, cholesterol cannot be converted into bile acids, but is instead converted into cholestanol and bile alcohol. Providing chenodeoxycholic acid exogenously has a negative feedback effect that reduces synthesis of bile acid, thus preventing accumulation of cholestanol.

In 1974, Setoguchi et al. first found that the decrease in bile acid synthesis in patients with CTX resulted from impaired oxidation of the cholesterol side-chain, which suggested that CTX is linked to a disorder of bile acid synthesis [6]. Patients with CTX lack mitochondrial sterol 27-hydroxylase (EC 1.14.13.15). This enzyme is located on the inner membranes of the mitochondria, is expressed in almost all cells of the body, and is an important enzyme in both the alternative and classic bile acid synthesis pathways [7,8]. Cholesterol 7α-hydroxylase (*CYP7A1*) is the rate-limiting enzyme in the classic pathway. In the alternative pathway, sterol 27-hydroxylase (*CYP27A1*) oxidizes cholesterol to 27-hydroxycholesterol, which is subsequently hydroxylated by oxysterol 7α-hydroxylase, leading in humans mostly to the formation of chenodeoxycholic acid (CDCA) [8-10]. A deficiency of sterol 27-hydroxylase leads to reduced production of CDCA and subsequently to upregulation of cholesterol 7α-hydroxylase. Upregulation of the rate-limiting enzyme in the classic bile acid pathway results in elevated levels of 7α-hydroxy-4-cholesten-3-one, an efficient precursor to cholestanol [3]. Most of the cholestanol accumulated in patients with CTX is derived from 7α-hydroxylated metabolites of cholesterol, 7α-hydroxy-4-cholesten-3-one being the most important [3,11]. Development and progression of CTX are secondary to the further efficient conversion of 7α-hydroxy-4-cholesten-3-one into cholestanol and bile alcohols by two different pathways [3,12] (Figure 1).

## General symptoms

Patients with CTX present diverse manifestations with multi-organ involvement and a broad range of neurological and non-neurological symptoms [13]. For example, intractable infantile-onset diarrhea and psychomotor retardation are common coexisting clinical features of CTX [14,15]. The mean age at onset of symptoms in patients with CTX is 19 years, but the average age at the time of diagnosis is 35 years (range 23–44), thus representing a diagnostic delay of 16 years (range 2–34) [5].

### Central nervous system involvement

Central nervous system symptoms and signs commonly present and sometimes constitute the initial manifestations in patients with CTX [16-22]. Epilepsy and Parkinsonism are the initial neurological features of CTX [19,20]. In a retrospective study involving 25 patients in Spain, Pilo-de-la-Fuente et al. divided the neurological manifestations into two main clinical subgroups, the classic form (cerebellar and supratentorial symptoms) and the spinal form (chronic myelopathy) [5]. The range of neurological features of CTX reported in the literature is broad; these features include intellectual disability, dementia, psychiatric symptoms (i.e., behavioral changes, depression, agitation, hallucination, and suicide attempts), pyramidal signs, progressive ataxia, dystonia, and palatal myoclonus [16,17,19-21,23-26].

### Ocular system involvement

Childhood-onset cataract is a typical sign of CTX [13-15]. This has been emphasized as an early symptom preceding neurological signs and tendon xanthoma, and is considered useful for early diagnosis. Cataracts and optic disk paleness are also the common ocular features in adults with CTX [21]. Other ocular abnormalities in CTX include retinal vessel sclerosis and cholesterol-like deposits [21,27].

### Cardiovascular system involvement

Premature atherosclerosis and cardiovascular disease have been reported among the multiple clinical manifestations of CTX [28]. Patients with CTX suffered from severe premature atherosclerosis in spite of normal serum cholesterol concentrations [29]. Blood lipid analysis in patients with CTX revealed dramatically high levels of 27-hydroxycholesterol and low levels of high-density lipoprotein cholesterol,which place patients with CTX at a high risk of suffering from cardiovascular disease [30].

### Skeletal system involvement

Osteoporosis and repeated bone fractures are also common clinical manifestations of patients with CTX. Low bone mass in the patient with severe gait disturbances increases the risk of accidental falls and bone fractures [31-33]. Federico et al. discovered that serum calcium, phosphate, and vitamin D metabolites in CTX patients were normal, but the total body bone mineral density was low and intestinal radiocalcium absorption was decreased [33]. However, the underlying pathogenesis is still unknown. There is no correlation between the severity of osteoporosis and biochemical parameters (i.e. cholestanol level, phenotypic data, and disability associated with neurological dysfunction) in patients with CTX [32].

### Pulmonary system involvement

**Figure 2:**
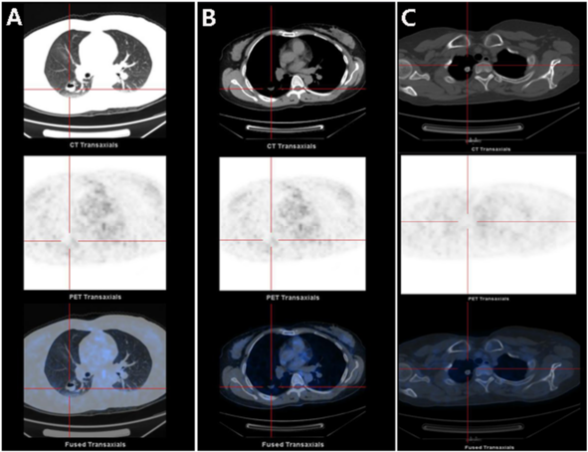
**Positron emission tomography (PET) imaging results in CTX: pulmonary system involvement.** PET reveals a cyst with a gas-fluid level (16 × 20 mm, **A** and **B**) and a high-density lesion (12 × 14 mm, **C**) in the lung.

Kawabata et al. discovered accumulations of foamy and giant cells engorged with cholestanol in bronchoalveolar lavage fluids and lung biopsy of patients with CTX, which demonstrated that the lungs are involved in CTX [34]. Patients with CTX with pulmonary involvement may have no clinical pulmonary symptoms (e.g., shortness of breath, chest distress) and no disturbance in pulmonary function tests [34]. Based on the results of positron emission tomography (PET), we found a high-density lesion (12 × 14 mm) and a cyst with a gas-fluid level (16 × 20 mm) in the lung of a patient with CTX (Figure 2). Pulmonary lymphangioleiomyomatosis was detected in a patient with CTX, but whether there is an association between these two rare diseases is unknown [35].

### Enterohepatic system involvement

In a large series of 32 patients with CTX studied by the Verrips et al., 50% had chronic and intractable diarrhea, which began in childhood [36]. Ninety-two percent of the patients with CTX in another large retrospective study in Spain had chronic diarrhea [5]. However, the gastro-intestinal examinations in many of the patients with diarrhea were normal [37]. Diarrhea in patients with CTX disappears a shortly after the start of CDCA therapy; however, the underlying mechanism is unknown [15,36,37]. This may be partly because bile alcohols produced in CTX are replaced by CDCA, which is an excellent micelle-forming bile acid, thereby improving fat and fat-soluble vitamin absorption and the attendant diarrhea. Some patients with CTX also develop neonatal cholestatic jaundice, cholecystic polypus, and gallstones [21,38].

### Peripheral nervous system and muscle involvement

Ginanneschi et al. revealed that 74.2% of patients with CTX (n =35) showed peripheral nerve abnormalities [39]. Demyelination and remyelination, features of axonal degeneration, can be found in patients with CTX [40]. Mild myopathic changes and ultrastructural abnormalities in mitochondria are observed in muscle lesions [41]. Of note, the presence of tendon xanthomas is not necessary for the diagnosis of CTX because not all patients have visible tendon xanthomas.

### Laboratory findings

The biochemical abnormalities in CTX include a plasma cholestanol concentration five- to ten-fold greater than normal (330 ± 30 μg/dL), a urine bile alcohol concentration of 14,000 ± 3,500 nmol/L, and a plasma bile alcohol concentration more than 500- to 1,000-fold greater than normal (8.48 ± 3.67 nmol/L). The biochemical abnormalities that distinguish CTX from other diseases with xanthomas include: high plasma cholestanol concentration, normal-to-low plasma cholesterol concentration, decreased CDCA level, and increased levels of cholestanol and apolipoprotein B in cerebrospinal fluid [42]. An elevated plasma 5-α-cholestanol concentration detected by gas chromatography–mass spectrometry (GC-MS) is a biomarker for CTX [43].

### Instrumental examinations

Transcranial magnetic stimulation (TMS) is a useful tool for detecting corticospinal tract damage and for evaluating improvements in pyramidal function after CDCA therapy [44]. In CTX, abnormalities are seen in neurophysiological examinations such as visual evoked potential (VEP), somatosensory evoked potential (SSEP), brainstem auditory evoked potential (BAEP), and nerve conduction velocity (NCV), tests that have been widely described [5,39,45-47].

### Imaging

Imaging studies have a significant role to play in prompt diagnosis.

**Figure 3:**
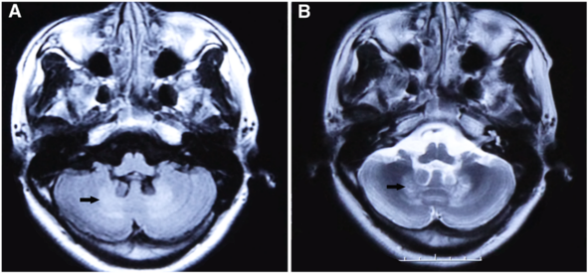
**MRI results: brain.** Brain MRI shows T1-weighted (**A**, arrow) and T2-weighted (**B**, arrow) hyperintensities in the dentate nuclei.

**Figure 4:**
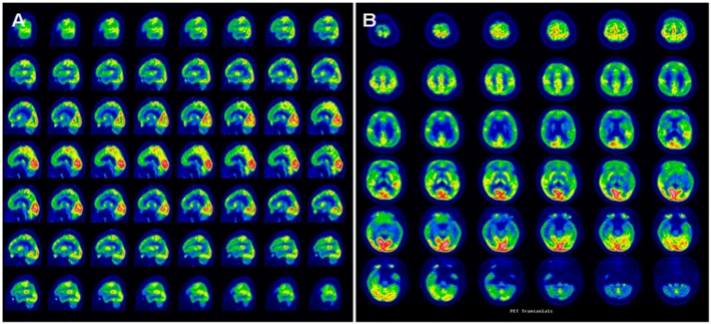
**PET imaging results: brain.** Remarkable abnormalities are seen in the basal brain metabolic rate in a patient with CTX. PET reveals hypometabolism in cerebral lobes (especially in the frontal and temporal lobes) in sagittal section **(A)**, and in axial section **(B)**.

First, the brain MRI reveals cerebellar atrophy, white matter signal alterations, and symmetric hyperintensities in the dentate nuclei [42,48-53] (Figure 3). Gray matter (GM) and white matter (WM) volume are diffusely decreased in patients with CTX [54,55]. In additional to conventional MRI, diffusion tensor imaging (DTI) and voxel-based morphometry (VBM) can provide complementary information about the involvement of GM and WM in CTX [54]. DTI is a useful tool for detecting white matter tract changes, because it is sensitive to water diffusion. VBM as an unbiased brain quantitative method that can be used to delineate volume losses in GM and WM [56]. Several previous magnetic resonance spectroscopy (MRS) studies revealed increased lactate and lipid peaks in FLAIR-hypointense lesions and decreases in N-acetylaspartate (NAA) peaks diffusely [49,51,57,58]. SPECT imaging reveals regional cerebral blood flow (rCBF) changes in multiple brain lobes before and after therapy, which might be a useful tool for monitoring the response to therapy in patients with CTX [54,59,60]. SPECT imaging can assess mitochondrial status and presynaptic dopaminergic function (using the special photographic developers ^99m^Tc-sestamibi and ^123^I-FP-CIT, respectively) associated with Parkinsonian symptoms in CTX [61,62]. Using ^18^F-6-fluoro-L-dopa for PET analysis, Kuwabara et al. found reduced uptake of dopamine into the putamen in a patient with both CTX and hemiparkinsonism, suggesting a dysfunction of presynaptic dopaminergic neurons, which is not classical in CTX [63]. The PET images exhibited remarkable differences in basal brain metabolic rate between patients with CTX and normal volunteers (Figure 4).

**Figure 5:**
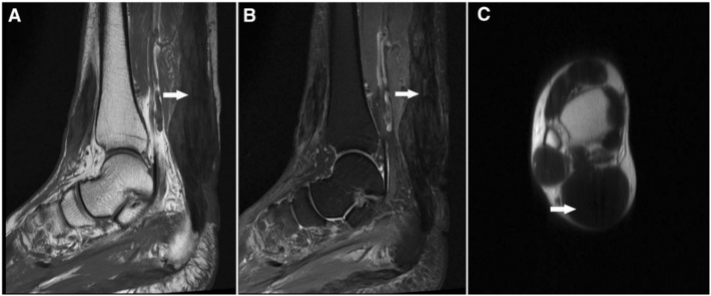
**5 MRI results: tendon.** MRI of both ankles. Sagittal long-T1-weighted image (**A**, arrow) and short-T2-weighted image (**B**, arrow), and axial long-T1-weighted image (**C**, arrow) of fusiform thickenings in the Achilles tendons in a patient with CTX.

**Figure 6:**
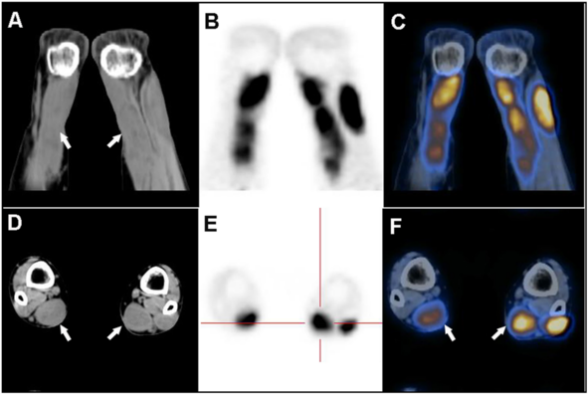
**PET imaging results: tendon.** Unusually high radioactivity is found in both Achilles tendons of a patient with CTX. PET shows abnormal soft-tissue thickening in a CT window in coronal section **(A)** and in axial section **(D)**, and unusually high radioactivity in Achilles tendons and adjacent regions in PET **(B and** **E)** and fusion windows **(C** **and F)**.

Second, MRI of both ankles shows fusiform thickening and heterogeneous signals [24] (Figure 5). Moreover, PET analysis using ^18^F-2-deoxy-2-fluoro-glucose shows abnormally high radioactivity in the Achilles tendons and adjacent regions (Standardized Uptake Value 8.7-13.6) (Figure 6).

Third, radiological images of the lung have been reported in several cases with CTX. The lesions in the lungs manifest as diffuse, nodular, infiltrated, and fibrotic shadows, which suggests interstitial pulmonary dysfunction or xanthomatous lesions [21,34,35].

### Pathology

**Figure 7:**
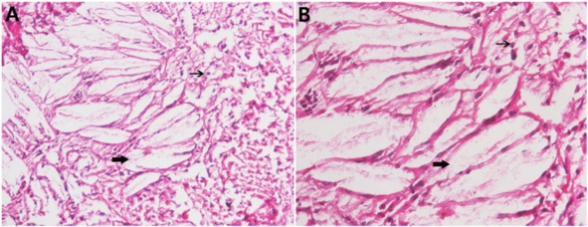
**Histology: tendon.** HE staining of the tendon masses reveals accumulation of xanthoma cells (fine arrows) and dispersed lipid crystal clefts (coarse arrows). **A**, 100×; **B**, 200 × .

Macroscopic section of the brain shows brain atrophy with multiple yellowish deposits in the plexus choroideus and in brain white matter [64]. Under microscopic examination, pathological findings in the central nervous system in CTX include multiple dispersed lipid crystal clefts and granulomatous lesions in the cerebellar hemispheres, demyelination and perivascular accumulation of foamy macrophages in the globus pallidus, and extracellular deposition of homogeneous myelin-like material in periventricular areas [52,64-66]. Demyelination, gliosis, and involvement of the long tracts of the spinal cord have also been described [52,67]. Nerve biopsy reveals primary axonal degeneration, demyelination, and remyelination [40,68-70]. The pathological findings from needle aspiration and autopsy of the lungs of patients with CTX reveal granulomatous materials, foamy cells, and intracellular accumulations of foreign bodies [34,35,71]. Histopathology of the tendon masses shows an accumulation of xanthoma cells and multiple, dispersed lipid crystal clefts [70] (Figure 7). Under electron microscopic examination, ultrastructural abnormalities are found in the lesions, including subsarcolemmal accumulation in mitochondria and swollen sarcoplasmic reticulum [46].

### Genetic analysis

The gene coding for sterol 27-hydroxylase was first located on the q33-qter interval of human chromosome 2 and mouse chromosome 1 by the group of Russell [1]. DNA sequence analysis of *CYP27A1* predicted a human sterol 27-hydroxylase consisting of a 33 amino-acid mitochondrial signal sequence followed by 498 amino acids [1]. Various mutations in all nine exons and in introns 2,4,6,7, and 8 of the *CYP27A1* gene have been described worldwide [8]. Fifty percent of mutations in *CYP27A1* have been detected in the region of exons 6–8, 16% in exon 2, and 14% in exon 4 [8,72]. Various mutation types, including missense (approximately 45%), nonsense (approximately 20%), splice site (18%), deletion (14%), and insertion (2%) have been detected in all nine exons of *CYP27A1* [2]. No genotype-phenotype correlations have been identified in CTX [2,5,73]. It is notable that *CYP27A1* was identified as a candidate gene for sporadic amyotrophic lateral sclerosis in a large genome-wide screening study [74,75].

## Differential diagnosis

### Sitosterolemia

Sitosterolemia (OMIM#210250), is a very rare inherited sterol storage disease caused by mutations in the adenosine triphosphate-binding cassette (ABC) transporter genes *ABCG5* and *ABCG8*, which are located on chromosome 2p21 and expressed at the membrane of enterohepatic cells [76-78]. Around 100 sitosterolemia cases have been reported in the literature [79]. Increased absorption and decreased excretion of plant sterols are the metabolic characteristics of sitosterolemia [22,80]. Clinical manifestations are characterized by extensive tuberous and tendonous xanthomas, premature atherosclerosis, hemolytic anemia, arthritis, and thrombocytopenic purpura [79,81,82]. Laboratory findings included high plasma phytosterol concentration and normal to mildly elevated plasma cholesterol concentrations [22,79]. Combined treatment with ezetimibe and a low plant-sterol diet can be effective in reducing plant sterol levels in the plasma, promoting xanthoma regression, improving the cardiovascular and hematological signs [79,83].

### Dyslipidemias

Familial hypercholesterolemia (FH; OMIM#143890) is an autosomal-dominant disease caused by a deficiency in low-density lipoprotein (LDL) receptor (ADH1) activity or in LDL-related genes (*ApoB* and *PCSK9*), which leads to obviously elevated LDL cholesterol (LDL-C) and triglyceride concentrations [10,84]. The prevalence of FH is estimated to be 1/300-500 (heterozygous type) worldwide [84,85]. The underlying pathogenesis of FH is the decreased clearance of apolipoprotein B-containing lipoproteins and their subsequent accumulation in multiple tissues [22]. The diagnostic criteria for FH in adults (>15 years of age) include hyper-LDL cholesterolemia (untreated LDL-C level ≥180 mg/dL), tendonous xanthomas (xanthoma on the backs of the hands, elbows, knees, etc.) or xanthoma tuberosum, and a family history of FH or premature coronary artery disease [84]. Familial dysbetalipoproteinemia may present with palmar crease xanthomas, but familial hypercholesterolemia presents with intertriginous xanthomas in children, and sitosterolemia and CTX present with tendonous xanthomas in adults [86]. Lifestyle modification and high-potency statins should be the first-line treatment and can delay the onset of coronary artery disease [84,87].

Neurologic symptoms and diarrhea, which are important features of CTX, are non-existent in patients with sitosterolemia or FH [22]. Note also that the morphology of xanthomatosis is variable. Depending on the clinical morphology, localization, development, and progression of this lipid storage disorder, xanthomas can present as either eruptive, tuberous, tendonous, or planar [10,86]. CTX can be distinguished from other lipid storage disorders by its specific clinical features such as childhood-onset cataracts, progressive neurologic symptoms, mild pulmonary insufficiency, increased plasma cholestanol levels, and the results of the SSEP assessment [10,22].

### Other inborn errors of bile acid metabolism

Other disorders of bile acid synthesis and metabolism include cholesterol 7α-hydroxylase deficiency (mutation in the *CYP7A1* gene), 3β-hydroxy-C_27_-steroid oxidoreductase deficiency,and 2-methylacyl-CoA racemase deficiency [88]. Patients with cholesterol 7α-hydroxylase deficiency have elevated serum cholesterol concentrations and are unresponsive to hydroxymethylglutaryl (HMG)-CoA reductase inhibitor therapy. Neonates with 3-β-hydroxy-C_27_-steroid oxidoreductase deficiency show hepatomegaly, mild steatorrhea, elevated serum ALT and AST, hyperbilirubinemia, and normal serum γ-glutamyl transpeptidase [89]. Defects in 2-methylacyl-CoA racemase have profound effects on both the bile acid and the fatty acid pathways. Patients may present with a sensorimotor neuropathy in adults and with a fat-soluble vitamin deficiency, hematochezia, and cholestatic liver disease in infants [90]. Bile acid therapy with cholic acid (10 to 15 mg · kg^−1^ · day^−1^) has also been proved effective in treating the above three metabolic disorders.

## Diagnosis

Early detection and diagnosis of CTX is crucial because early and long-term treatment of CTX with CDCA improves neurological symptoms and even reverses the progression of the disease [5,23,38,53,77]. However, an obvious delay between symptom onset and diagnosis is prevalent [5,38].

The diagnosis of CTX is mainly based on clinical findings, biochemical testing, neuroimaging, and molecular genetic analysis. A diagnosis of CTX should be considered for patients with xanthomas and neurological symptoms starting in childhood. It should be noted that the symptoms might also start in adulthood [91]. The biochemical abnormalities of patients with CTX in the laboratory examination include elevated plasma cholestanol level and increased levels of bile alcohols in urine associated with a diminished biliary concentration of chenodeoxycholic acid [22]. An elevated plasma level of cholestanol is a feature of CTX [10,22,43]. It was reported that the serum levels of 7-α-hydroxy-4-cholesten-3-one and cholesta-4,6-dien-3-one in patients with CTX were 100 times higher than normal [92]. A new and sensitive multi-analyte blood test with liquid chromatography–electrospray ionization-tandem mass spectrometry (LC-ESI-MS/MS) methodology can be used to quantify plasma ketostetrol bile acid precursors such as 7-α-hydroxy-4-cholesten-3-one and 5-α-cholestanol [43]. With the development of genetic analysis, MRI and other neuroimaging technologies are not crucial for the diagnosis of CTX, but could provide complementary clinical information [93].

On the basis of a pool analysis of a screened international CTX series, Mignarri et al. developed an effective suspicion index for early diagnosis composed of weighted scores related to indicators such as family history and systemic and neurological characteristics [38]. The indicators were classified as very strong (score = 100), strong (score = 50), or moderate (score = 25). Childhood-onset cataracts, diarrhea, and neonatal cholestatic jaundice in combination with neurological features and dentate nucleus abnormalities in MRI were demonstrated to be strong indicators. Tendon xanthomas were considered very strong indicators. Plasma cholestanol examination with a total score ≥100 is requisite, as is *CYP27A1* gene analysis with a total sore ≥200 or the existence of one very strong or four strong indicators. Using this efficient diagnostic tool, the investigators achieved a diagnostic age in their study of only 10.6 ± 9.8 years, which compares favorably to the previous average age at diagnosis of 35 years (*p* <0.01) [38].

## Management and prognosis

The management of CTX includes replacement therapy, surgery, and other symptomatic therapy. Our knowledge of the pathogenesis of CTX suggests that replacement therapy involving bile acid supplementation can restore bile acid synthesis by reducing plasma cholestanol levels and eliminating bile alcohols. Moreover, the benefits could be enhanced by administration of HMG-CoA reductase inhibitors [22,94]. The potential mechanism of bile acid therapy may be exogenous inhibition of bile acid production by activating the bile acid negative feedback mechanism. This would inhibit production of the intermediate 7-α-hydroxy-4-cholesten-3-one, thereby normalizing cholestanol concentration and preventing the accumulation of cholestanol in tissues.

Replacement therapy involves administration of bile acids such as CDCA, ursodeoxycholic acid (UDCA), cholic acid, or taurocholic acid [95-97]. Compared to administration of UDCA or taurocholic acid, CDCA treatment (750 mg/d) is the therapy of choice for treating the neurological and non-neurological symptoms of CTX, but cholic acid is also efficient for non-neurological symptoms [39,41,44,73,96]. Bile acid therapy with cholic acid has also proven its effectiveness in treating other lipid metabolic disorders [88]. Administration of CDCA (750 mg/d) or cholic acid can normalize plasma cholestanol and improve non-neurological symptoms in many individual CTX cases, but only CDCA can improve the neurological symptoms in patients with CTX [5,23,95,97]. Long-term replacement therapy with CDCA can increase bone mineral content and improve intestinal absorption of vitamin D by activating the bile acid negative feedback mechanism [32]. Combination therapy with CDCA (300 mg/d) and pravastatin (10 mg/d) can improve lipoprotein metabolism, inhibit cholesterol synthesis, and reduce plasma levels of cholestanol and plant sterols [98]. The efficacy of treatment with HMG-CoA reductase inhibitors alone is controversial, and some adverse effects such as hepatic dysfunction and rhabdomyolysis may be observed [98,99]. Other possible treatments that lack reliable clinical validation include vitamin E supplementation, low-density lipoprotein apheresis, and liver transplantation [5,21,24,100]. Surgical excision of bilateral tendon may worsen the gait imbalance and cannot prevent the deterioration of neurologically affected patients [22]. Due to the diverse manifestations and signs of CTX, symptomatic therapy is essential: antidepressant medication in case of depression [24], antiepileptic therapy in case of convulsive seizures [19], levodopa in case of parkinsonism, and botulinum toxin in case of dystonia [61,101,102].

Beginning treatment with bile acid therapy as early as possible is crucial for preventing neurological damage and deterioration in patients with CTX [5,21,23,32,95,103]. In a large series of 25 patients with CTX, 60% of patients continued to deteriorate and 20% died in spite of the long-term administration of CDCA, but survival was related to age at diagnosis [5]. Ginanneschi et al. revealed that CDCA treatment improved nerve conduction velocity and promoted myelin synthesis in nerve fibers with residual unaffected axons in a series of 35 patients with polyneuropathy, and the therapeutic effect depended largely on the extent of irreversible structural damage [39]. Neurophysiological follow-up research might be recommended when patients with CTX present clinical symptoms of peripheral neuropathy [45].

## Discussion and unresolved questions

Monitoring plasma cholestanol levels can be used to assess the biochemical effects of CDCA and cholic acid in patients with CTX before and after treatment. However, serum cholestanol level has no correlation with clinical features [44]. A possible explanation is that increased cholestanol level is not the only factor important for pathogenesis in CTX. Further studies are required to understand any other underlying mechanisms and to provide reasonable explanations. Cerebral WM lesions and cerebellar vacuolation have been described in a patient with CTX with progressive ataxia [49,75]. White matter changes in patients with CTX suggest the coexistence of demyelinating and axonopathic lesions in CTX [45]. Moreover, further studies are needed to discover why some patients with CTX develop WM lesions in the brain.

Many clinical studies of CTX have been published, but basic animal research on the pathogenesis of CTX is still not commonly reported. The *CYP27A1* gene knockout mice do not present with xanthomas in brain or tendon. However, mice may be able to compensate for the loss of the alternative bile acid synthesis pathway [8,104]. Transgenic mice overexpressing the *CYP27A1* gene do not show increased synthesis of bile acid [8]. The reason for the obvious difference between the animal models and humans with CTX is not known with certainty. The relationship between the deposition of cholestanol and the development of xanthomas deserves further study.

## Conclusion

CTX is an inherited lipid metabolic disorder with diverse manifestations. The classical symptoms and signs, namely elevated levels of cholestanol and bile alcohols in serum and urine, cranial magnetic resonance imaging, and mutation in the *CYP27A1* gene, confirm the diagnosis. Patients with CTX have an average diagnosed age of 35 years and a diagnostic delay of 16 years. Early diagnosis and long-term treatment with CDCA (750 mg/d) can improve neurological symptoms and contribute to a better prognosis.

## Consent

Written informed consent was obtained from the patient for the publication of this report and any accompanying images.

## Abbreviations

CTX: Cerebrotendinous xanthomatosis
*CYP7A1*: Cholesterol 7α-hydroxylase
*CYP27A1*: Sterol 27-hydroxylase
CDCA: Chenodeoxycholic acid
GC-MS: Gas chromatography–mass spectrometry
TMS: Transcranial magnetic stimulation
VEP: Visual evoked potentials
SSEP: Somatosensory evoked potentials
BAEP: Brainstem auditory evoked potentials
NCV: Nerve conduction velocity
MRI: Magnetic resonance imaging
DTI: Diffusion tensor imaging
VBM: Voxel-based morphometry
GM: Gray matter
WM: White matter
NAA: N-acetylaspartate
PET: Positron emission tomography
ALS: Amyotrophic lateral sclerosis
ABC: Adenosine triphosphate-binding cassette
LDL: Low density lipoprotein
LDL-C: LDL cholesterol
FH: Familial hypercholesterolemia
LC-ESI-MS/MS: Liquid chromatography–electrospray ionization-tandem mass spectrometry
UCDA: Ursodeoxycholic acid
